# The knowledge about patient safety among undergraduate nurse students in Cyprus and Greece: a comparative study

**DOI:** 10.1186/s12912-021-00610-6

**Published:** 2021-06-25

**Authors:** Maria Dimitriadou, Anastasios Merkouris, Andreas Charalambous, Chrysoula Lemonidou, Evridiki Papastavrou

**Affiliations:** 1 Department of Nursing, School of Health Sciences, Cyprus University of Technology, 15 Vragadinou Str, 3041 Limassol, Cyprus; 2 Department of Nursing, National and Kapodistrian University, 123 Papadiamadopoulou str, 11517 Goudi, Athens, Greece

**Keywords:** Nursing students, patient safety, undergraduate education, curriculum

## Abstract

**Background:**

The Patient safety movement contributed to the reduction of preventable adverse events associated with health care. Although patient safety issues have received the attention of educators in the health care studies, there is evidence that in nursing education and the associated curricula it is not well-incorporated. This may not allow students to acquire scientific knowledge and develop strong competencies to assure patient safety throughout their professional life. The aım of the study was the exploration of the undergraduate nursing student perspectives regarding knowledge received during their training about patient safety-related issues.

**Methods:**

A descriptive comparative study was conducted with three and four-year undergraduate nursing students from the Cyprus Republic (*n* = 243) and Greece (*n* = 367). All students were surveyed using the Health Professional Education Patient Safety Survey (H-PEPSS) to describe students’ knowledge in the classroom and clinical setting.

**Results:**

Students’ Knowledge about patient safety was expressed significantly higher (*p* < 0.001) in the classroom (mean = 4.0) than the clinical setting (3.7) (1–5 scale). The knowledge in the dimension “clinical aspects” received the highest score and “working in teams” received the lowest. Also, differences were recorded between countries wıth Cypriot students reporting hıgher level of knowledge than the Greek students in most of the dimensions.

**Conclusıon:**

The findings revealed the gap between theory and practice and the need for collaboration between the two settings. Also, students reported relatively higher knowledge with regards to the technical aspects of patient safety. Still, they were less knowledgable about the sociocultural aspects of the patient, such as working in teams.

## Background

The fundamentals of good nursing are reflected in Florence Nightingale’s (1896) quoted words “First, do no harm” and are expressed in codes of ethics and responsibility [1, 2]. Today healthcare organisations face many challenges in keeping and promoting safe care for patients due to resources shortage, the increasing demand for care, technological advancements and shifting population demographics. Despıte these challenges, patient safety should be considered a human-right issue and health care systems should be committed to all their patients [1].

Globally, it has been estimated that one out of ten patients experienced safety issue(s) while receiving hospital care; this represents the 14th cause of the global disease burden [3]. A survey among European citizens showed that half of the respondents felt that they might be harmed while receiving healthcare and a high percentage of the respondents claimed that they or a family member had experienced an adverse event during healthcare [4]. The economic burden of unsafe care is estimated as the annual cost of common adverse events (i.e. pressure ulcers, hospital-associated infections, thromboembolism, medication error) being equivalent to hiring 2,000 General Practitioners or 3,500 hospital nurses [5]. It is alarming to realise that many adverse events are preventable, and up to 28 USD billion has been saved in 5 years by improving safety in hospitals [5]. These facts corrode public trust and build vast health, financial and ethical burden on the healthcare systems and the broader society.

‘Patient safety’ is defined as the prevention of unnecessary harm to a patient during the process of health care and the reduction of the risk of unnecessary injury, associated with health care, to an acceptable minimum [6]. Patient safety is the outcome of both system effectiveness and individual performance design to minimise the risk of injuries to patients from the care that is intended to help them [7]. That encompasses shifting from the position of thinking patient safety as a technical issue to the position of system-related factors and the involvement of many individuals [8]. These factors are referred to as non-technical or sociocultural skills of patient safety [9, 10]. Non-technical skills are defined as *‘a set of social and cognitive (analytical and personal behaviour) skills that support high-quality, safe, effective, and efficient interprofessional care within the complex healthcare system’ (p. 6)* [11]. Key elements, included in sociocultural skills, are effective communication, teamwork, skills of recognising and managing risky situations, optimising human and environmental factors and contributing to a culture aware of the importance of reporting and learning from incidents [10].

It is important to note that nurses are the largest group of health providers as they make up more than 50 % of the practicing health workforce and they have a central role in protecting patient safety, as they are the providers with the most prolonged and most direct patient contact [12]. Thus, nurses, more than any other health professionals, are likely to recognise, prevent, and correct malpractice in workflow and communication [12].

Undergraduate nursing education is an important starting point in advancing patient safety in the domains of knowledge, attitude, and skills in preparing future nurses [10, 12–14]. Students are considered an integral and indispensable component of the health care system, contributing to safety care, highlighted in the statements “*fresh pair of eyes, keen to learn.and .the safety leaders of the future*“[2]. As such, exposure to the concept of patient safety early in undergraduate education is encouraged by two facts: firstly, more preventable errors are made by nurses shortly after graduation than later in their career [15], and secondly, the new graduates bring current evidence-based theory and a fresh vigour to the workplace[16]. To a degree, nursing education is associated with patient mortality [17–19] and the level of nurse-reported adverse events [20, 21]. Today a gap of evidence about the extent and type of the role of nursing education in patient safety improvement [12, 22], as well as the perceived confidence of undergraduate nursing students in specific patient safety areas still exist [23].

The landmark in advancing patient safety as a priority and quality indicator was the United State’s report “Error is human”,[24] and the report “*An Organization With a Memory”* launched later by the United Kingdom [25]. Since then, healthcare professional education has been seen as one of the most crucial improvement intervention for reducing the risk of harm in order students, as the future workforce, to understand the nature of risk in health care and the need of bolstering systems [10, 24, 26, 27]. Traditionally, health care professional curricula focus on clinical skills, such as diagnosis, treatment, medication administration and keeping aseptic techniques [12, 28]. However, unintended patient harm is predominantly a consequence of system failure [8]. For that, educational and organizational challenges extended the Individual responsibility further to include effective teamwork, information sharing, a collaboration between professions and respect for each other’s roles and perspectives [22] in order patient safety learning to be delivered in an integrated way.

Patient safety awareness has emerged in undergraduate education, aiming to change organisational culture and recommend teaching and learning skills related to patient safety. At an international level, the World Health Organization (2011) has developed “The Multi-Professional Patient Safety Curriculum Guide”. In Europe, the European Network for Patient Safety [26] project encapsulated the Council of European Union recommendations gıven in 2009/C 151/01 and raised awareness of integrating patient safety in the health professıonals’ curricula. This approach went beyond formal learning in the classroom, addressing guidelines in improving understanding in clinical settings. Finally, at a natıonal level, regulatory bodies have provıded a framework for learning interventions [10, 29–31]. All of them focus on the development of professıonal competencies and place patient safety beyond the actual technical provision of care (i.e. administration of medicine) to the improvement of the system that underpins practice. They identified human factors and the way these factors relate and interact within the system as a significant element of patient safety domain. The problem is that the framework contexts about patient safety principles are not domain-specific [32]. So, some additional knowledge relevant to the nursing specialisation (i.e.empowerment, psychological support and interprofessional relationships) is recommended [33].

Today, two decades later, patient safety education for health professionals is the least implemented among all the initiatives [14, 34]. Literature has identified that teaching patient safety to undergraduate nursing students is a necessity, however, there is still no consistency in the teaching methods nor an agreement on which areas to focus or prioritise. Patient safety remains a “hidden element” within the curriculum over the years [14, 35–41]. It is incorporated into an already overwhelming nursing curriculum slowly and somewhat sporadically [12, 39, 40, 42]. This, therefore, leaves students little opportunity to clarify and understand non-technical human factors role over technical competencies in keeping patient safe[43].

Earlier evidence indicated that students felt more confident in technical aspects than the sociocultural aspects of patient safety [9, 44–47]. For example, students reported less knowledge regarding teamwork issues, especially during their clinical placements [35, 46], although they valued teamwork ıncludıng patient participation in health care planning [48]. However, this evidence derived mainly from Canada, USA and Australia and partly from Europe (e.g. the UK, Finland and Italy) [32, 49–53]. Furthermore, students’ knowledge about patient safety beyond the formal classroom in the actual workplace was limited [54, 55].

Students felt that more class time was spent on teaching pathophysiology and treatment over nursing interventions [12]. As a result, not enough time was left to discuss nursing care and patient safety issues. Consequently, students considered themselves unsafe and not knowledgeable enough regarding patient matters in real practice [39, 40, 51]. Other evidence showed lack of faculty familiarity in teaching such courses [40] and the lack of educators’ confidence or interest to integrate patient safety in the curriculum [12, 29, 56]. A qualitative study has found that the WHO 11 topics were either not addressed at all (i.e. human factors) or were taught in a manner that failed to link patient safety [33].

Similarly, a European study, under the COST Action project RANCARE (https://www.cost.eu/actions/CA15208), across 27 countries, has found that safety ıssues were not included as a separate module. Even if they were indeed included in some syllabi, they were not taught as a stand-alone topic but were rather dispersed across the curriculum in several other subjects giving the ımpressıon of limited ımportance. Also, the above study identified differences in nursing education across and within the countries examined [14].

Reconstructing nursing education is challenging. The growing literature indicates a gap in knowledge, about where and how patient safety is effectively taught ın the pre-registration nursing program and about the level of knowledge students should obtain in specific patient safety areas [14, 55, 57]. Evaluatıon of nursıng students perceptıons on what they have learned about patient safety in both the classroom and the clinical practice could be a starting point, especially in the case where these students come from different nationalities and cultures and their viewpoints are brought to a bear [26, 34]. Studying multiple students perceptions is the best way for educators to understand the weaknesses and omission of the education system. Under the sight of the lımıted evidence in the perceived competence in patient safety in Europe, the data of the current study will add value to national efforts as well as the European collaboration to the process of designing, developing, delivering and evaluating a learning intervention either in class or the workplace. To our knowledge, this is the second comparative study between countries focusing on undergraduate nursing students’ perceptions of patient safety issues [50, 51].

In short, a nursing education description in Cyprus and Greece follows. Training of nurses in Cyprus and Greece conforms to the EU standards for mutual recognition of qualifications [58]. The duration of undergraduate nursing studies is four years covering 240 ECTS (European Credit Transfer and Accumulation System). Cyprus has only the option of a university degree graduation. Regarding the clinical practice, two supervision models have been adopted, that of the Nurse educator and the mentor. The mentor and the students are both supernumeraries in a ration 1:5 [59]. In Greece, at the time of data collection, there were two levels of higher nursing education (university and technological degree) and vocational education for nursing assistants (two-year studies). During clinical practice, students are trained either by nursing staff (mainly graduated from the university) or by graduate or doctoral students with clinical experience, whereas at times nursing professors visit students for ensuring that learning outcomes are fulfilled.

## Method

### Aim

The aim of the study was the exploration of the nursing student perspectives about patient safety knowledge and the nursing curricula among the populations of Greece and Cyprus.

### Study desıgn, setting and participants

A descriptive – comparative study. The target population of the study included all three and four-year students enrolled in an undergraduate nursing program as regulated by the European directive 2013/55/EU [58] in both countries. Particularly, in Cyprus, 229 students, from the 4 universities (1 public and 3 private) offering nursing bachelor program and in Greece, 381 students, from the biggest university of Greece offering nursing bachelor program were enrolled. The selectıon of Cyprus and Greece was based on the continuous collaboration of the two countries in educational matters, shared historical roots, tradition and culture, as well as the fact that they are both Greek-speaking countries. The selection of all three and four-year students was because they had completed several clinical placements and spent longer periods in the clinical practice than students of the other years. Also, senior students had focused more on non-technical skills, like leadership and guidance than the junior ones who had been directed toward essential clinical nursing skills [60]. The questionnaires were administered to all the students during their last classroom lesson between May and June 2018. Νo currıculum changes occurred over the study perıod.

### Study instrument

The study used the Health Professional Education in Patient Safety Survey (H-PEPSS) developed by Liane R. Ginsburg [9]. The instrument was developed to reflect specific patient safety educational outcomes regarding ‘non-technical skills’, as well as the gap between classroom knowledge and clinical competence[52, 61]. The questionnaire includes 38 items divided into 3 sections (Secs. 3, which explores the comfort of speaking up (6 items) hasn’t been included in the present study): The 1st section is composed of 27 items, divided into 7 dimensions: one dimension focuses on clinical safety issues (technical aspect) (4 items) and six dimensions focus on the sociocultural aspects of patient safety, proposed by the Canadian Patient Safety Institute (CPSI) [10]. These are: (a) Contributing to a culture of patient safety (4 items); (b) Working in teams for patient safety (6 items); (c) Communicating effectively for patient safety (3 items); (d) Managing safety risks (3 items); (e) Optimising human and environmental factors (3 items); and (f) Recognising, responding to and disclosing adverse events and close calls (4 items). Students were asked to indicate their agreement for each item regarding contents learned in the classroom and during their clinical experience with a separate score on a 5-point Likert scale ranging from 1= ‘strongly disagree’ to 5= ‘strongly agree’. The 2nd section is composed of five items focusing on how broader PS issues are addressed in health professional education. In both sections, each item is reported as a statement, scored on a 5-point Likert scale ranging from 1= ‘strongly disagree’ to 5= ‘strongly agree’. Higher scores represent higher levels of self-perceıved students’ knowledge about patient safety, in the specific areas. The psychometric properties of the instrument H-PEPSS were examined by several researchers [9, 44, 45, 52, 62] who concluded that it is a reliable (Cronbach’s a showed > 0.72) and a valid instrument, capable of evaluating competencies in patıent safety perceived by undergraduate nursing studıes. Demographic characteristics, including age, gender, year of study and country were also collected.

### Instrument translation

In order to verify the cross-cultural adaptation of the H-PEPSS a forward- backward translation, cultural and linguistic adaptation and a pilot study were attended, followıng the guide of the World Health Organızatıon process of translatıon and adaptatıon of ınstruments [63]. Firstly, a native English-speaking academic nursing staff and a certified translator translated independently the H-PEPSS from English into Greek. Then the two translators along with the two authors (MD, EP) proofread the translated instrument and agreed on the 1st draft. Then, two native English nurses, a doctoral degree holder and a doctoral degree candidate, back-translated the 1st Greek H-PEPSS draft. Subsequently, a new commıttee, consisting of two translators and three experts ın nursıng educatıon from Greece and Cyprus, compared the back-translation to the original, wıth emphasis on the conceptual and cultural, rather than the linguistic, equivalence. Then, a pilot study was conducted involving a convenience sample of 60 newly graduate nursing students. The students evaluated the fluency, the readability and the comprehensibility of the items. Finally, the appropriate adaptations were made according to the received feedback.

### Ethical considerations

 Approval was asked by the Cyprus National Bioethics Committees (CNBC EP: 2018.01.61) and the Ethics Committees of each university. Also, permission to use the H-PEPSS questionnaire was given by the author [9] vıa emaıl. The participants were informed in writing about the purpose of the study. Completing and returning the questionnaire was considered as consent to participate in the study. Fınally, confıdentıalıty as well as anonymity were assured, and the data was stored in a manner compliant with data protection regulations [64].

### Data analysis

SPSS 21.0 software (SPSS Inc., Chicago, IL) was used to perform descriptive and inferential statistical analyses. No imputation was done for missing data as was < 1 % observed at random. Statistically significant results were considered with p-value < 0.05. Demographic, class, and clinical setting-related data were summarised using descriptive statistics, mean (± standard deviation) calculated in the range of 1 to 5. Patient safety scores for each domain were calculated by averaging the items. Paired t-tests were performed to identify significant differences in patient safety competencies between the classroom and clinical scores, and independent samples t-tests between countries and year of studies.

## Results

### Demographic characteristics

Four-hundred and eighty-six students responded in the study (197 from Cyprus, and 289 from Greece) out of 610, giving a response rate of 79.5 % of the total participants. The majority were female (78.2 % Cyprus, 87.2 % Greece. Among the participants from Greece, 130 (45 %) and 159 (55 %) were 3rd and 4th -year students, respectively. Cypriot students attended a range of institutions and 104 (52.8 %), and 93 (47.2 %) were three and four-year students, respectively.

### The perceived knowledge in patient safety domains in the classroom and the clinical setting

As shown in Table 1, the internal consistency of the overall scale (Cronbach’s alpha) demonstrated an excellent internal consistency index a = 0.95 and a = 0.96 for the classroom and clinical setting, respectively. For the classroom dimensions, Cronbach’s alpha ranged from 0.89 (communicating effectively) to 0.85 (Understanding human and environmental factors) while for the clinical dimensions it ranged from 0.92 (working in teams) to 0.87 (Recognise and respond to an adverse event). The overall mean score was 4.0 ± 0.6 in the classroom setting, and 3.7 ± 0.8 in the clinical setting, from a range of 1 to 5. Nursing students exhibited the highest mean score in what they learned about, ın the *clinical safety* dimension ın both the classroom (4.4 ± 0.7) and the clinical setting (3.9 ± 0.9). The least knowledge was reported in “working in teams with other professionals” in both settings.

**Table 1:** Students’ perspectives of knowledge in patient safety dimensions (1^st^ section of the questionnaire- HPEPSS) in the classroom and clinical practice in both countries (Cyprus and Greece (*n* = 486) range = 1–5)

Patient safety dimensions	Classroom	Clinical	Paıred samples t-test	
Cyprus and Greece (*n* = 486)	Mean (SD)	Mean (SD)	t	*p*-value*
**Overall Scale**	4.0 (0,6)	3.7 (0,8)	10.507	< 0.001
Clinical Safety	4.4 (0,6)	3.9 (0,9)	11.693	< 0.001
Working in teams	3.7 (0,8)	3.5 (0,9)	6.808	< 0.001
Communicating effectively	4.1 (0,8)	3.8 (0,9)	9.039	< 0.001
Manage Risk	3.9 (0,9)	3.7 (1,0)	6.108	< 0.001
Human and environmental factors	3.9 (0,9)	3.7 (1,0)	5.428	< 0.001
Adverse events	3.9 (0,8)	3.6 (0,9)	7.212	< 0.001
Culture of safety	4.1 (0,8)	3.8 (0,9)	7.601	< 0.001

*Sig. (2-tailed)

### Differences in students’ perceptions between the year of study and countries.

Regarding the year of studies, 4-year students (irrespective of country of origin) exhibited higher scores in all HPEPSS dimensions when compared to 3-year students. Similarly to the comparison of means between countries, the highest score was reported in “clinical safety” and the lowest in “working in teams”. Also, in most of the dimensions, significant differences (*p* < 0.05) between the three and four-year students were identified. Three dimensions in the classroom setting and five dimensions in the clinical setting were found statistically significant (*p* < 0.05) with 4 -year students having a higher level of agreement when compared to three-year students (Table 2).

**Table 2:** Differences in Students’ perspectives of knowledge in patient safety dimensions between year of studıes and countries (range = 1–5)

Patient safety dimensions	Cyprus (*n* = 197)	Greece (*n* = 289)	Independent samples t-tests		3rd year (*n* = 234)	4th year (*n* = 252)	Independent samples t-tests	
	Mean (SD)	Mean (SD)	t	*p*-value*	Mean (SD)	Mean (SD)	t	*p*-value*
**Classroom setting**								
Clinical Safety	4.7 (0.5)	4.2 (0.7)	9.745	< 0.001	4.3 (0.7)	4.5 (0.6)	−3.216	0.001
Working in teams	4.1 (0.7)	3.5 (0.8)	9.343	< 0.001	3.7 (0.8)	3.8 (0.8)	−1.405	0.160
Communicating effectively	4.5 (0.6)	3.8 (0.8)	10.175	< 0.001	4.1 (0.8)	4.1 (0.8)	0.371	0.711
Manage Risk	4.3 (0.7)	3.6 (0.8)	9.957	< 0.001	3.8 (0.8)	4.0 (0.8)	−2.291	0.022
Human and environmental factors	4.3 (0.8)	3.7 (0.8)	8.202	< 0.001	3.9 (0.8)	3.9 (0.9)	−0.285	0.776
Adverse events	4.2 (0.7)	3.6 (0.8)	8.856	< 0.001	3.8 (0.8)	3.9 (0.9)	−1.361	0.143
Culture of safety	4.3 (0.6)	3.8 (0.7)	6.649	< 0.001	4.0 (0.8)	4.1 (0.7)	−2.654	0.008
**Clinical setting**								
Clinical Safety	4.5 (0.6)	3.5 (0.8)	13.107	< 0.001	3.8 (0.9)	4.0 (0.9)	−1.968	0.045
Working in teams	4.0 (0.8)	3.2 (0.8)	11.487	< 0.001	3.4 (0.9)	3.6 (0.9)	−1.790	0.075
Communicating effectively	4.3 (0.8)	3.4 (0.8)	11.232	< 0.001	3.7 (0.9)	3.8 (0.9)	−2.009	0.045
Manage Risk	4.1 (0.8)	3.3 (0.9)	9.914	< 0.001	3.5 (0.9)	3.8 (0.9)	−3.287	0.001
Human and environmental factors	4.0 (0.9)	3.4 (0.9)	6.411	< 0.001	3.6 (0.9)	3.8 (0.9)	−2.287	0.023
Adverse events	4.0 (0.7)	3.3 (0.8)	8.807	< 0.001	3.5 (0.8)	3.8 (0.9)	−3.442	0.001
Culture of safety	4.1 (0.8)	3.6 (0.9)	7.039	< 0.001	3.7 (0.9)	3.9 (0.8)	−3.463	0.001

*Sig. (2-tailed)

When comparing the Cypriot to the Greek students, as shown in Table 2, significant differences < 0,001 were found across all dimensions in both settings, with Cypriot students (mean 4.0-4.7 including both settings) reporting a higher level of perceived knowledge. Both, Cypriot and Greek students reported the highest knowledge on the dımensıon “clınıcal safety” in both settings and the least knowledge on the dimension “Working in teams with other health professionals” again in both settings. Remarkably, the difference in the mean scores between the two countries in the clinical setting ranged from 0,6 to 1, with the dimension of clinical safety having the highest mean dıfference (Table 2).

### Differences in students’ perceptions on “broader aspects of patient safety”

Table 3 shows that the level of agreement of the Cypriot student perceptions of how broader patient safety issues were addressed within their educational program was significantly different (*p* < 0.05) from that of the Greek students. Comparing students’ country of origin and years of study, the highest mean was expressed in the item “I gained a solid understanding that reporting adverse events and close calls can lead to change and can reduce reoccurrence of events”. As for the Cypriot students, the least rated learning experience was on the item “‘System’ aspects of patient safety are well covered in our program” whereas, for the Greek students, it was the item “My scope of practice was very clear to me”. The item “There is consistency in how patient safety issues are dealt with by preceptors in the clinical setting” followed as the second least-rated learning experience for both Cypriot and Greek students. The t-test revealed a statistically significant difference between the two countries of study in all items. Concerning the years of study, in most of the items, there was no significant difference between three and four-year students (*p* > 0.05) except of two items “I had sufficient opportunity to learn” and “System aspects of patient safety are well covered in our program” (Table 3).

**Table 3:** Dıfferences ın Students’ perceptions about broader aspects of patient safety (range = 1–5)

	Cyprus (*n* = 197)	Greece (*n* = 289)	Independent samples t-tests		3rd year (*n* = 234)	4th year (*n* = 252)	Independent samples t-tests	
Broader aspects on patient safety	Mean (SD)	Mean (SD)	t	p *	Mean (SD)	Mean (SD)	t	p *
Total	4.0 (0.6)	3.3 (0.6)	1	0.000	3.5 (0.7)	3.7 (0.7)	−3.099	0.002
My scope of practice was very clear to me	3.9 (1.0)	2.9 (1.1)	10.978	< 0,001	3.2 (1.2)	3.4 (1.2)	−1.574	0.116
There is consistency in how patient safety issues are dealt with by clinical educator in the clinical setting	3.8 (0.9)	3.0 (1.0)	9.566	< 0,001	3.3 (1.0)	3.4 (1.0)	−1.205	0.229
I had sufficient opportunity to learn and interact with members of interdisciplinary teams’	4.0 (0.9)	3.5 (1.1)	5.776	< 0,001	3.5 (1.0)	3.9 (1.0)	−4.226	0.001
I gained a solid understanding that reporting adverse events and close calls can lead to change and can reduce reoccurrence of events	4.3 (0.7)	3.8 (0.9)	6.096	< 0,001	3.9 (0.9)	4.0 (0.8)	−1.228	0.220
‘Patient safety is well integrated into the overall program	4.0 (0.9)	3.4 (0.9)	7.78	< 0,001	3.6 (0.9)	3.7 (1.0)	−1.547	0.123
Clinical aspects of patient safety are well covered in our program (e.g. hand hygiene, medication)	4.3 (0.8)	3.5 (1.0)	9.657	< 0,001	3.8 (1.1)	4.0 (1.0)	−1.794	0.073
“System” aspects of patient safety are well covered in our program (e.g. organization, management or the work environment)	3.4 (0.9)	3.2 (1.0)	8.021	<0,001	3.3 (1.0)	3.7 (0.9)	−3.999	0.001

*Sig. (2-tailed)

## Discussion

In thıs study, overall, students reported that they were more confident in their knowledge about patıent safety gained in the classroom setting than in the clinical setting. These findings are similar to some studies [37, 38, 44, 46, 61, 65, 66], although other studies reported mixed results, with reported clinical scores being higher than the class scores in more than half of the dimensions [9, 45, 65]. Classroom ıs perceived as a safe environment of learning about working in multi-professional teams, understanding the system-based nature of patient safety problems [50] and being more confıdent to speak up [66, 67]. As 50 % of the student programme is completed in clinical practice, its contribution to professional socialisation is identified. However, students feel unprepared when entering a clinical practice environment. If education in academic settings is too theoretical, nursing students may consider themselves as unsafe for patient care [12, 39]. In clinical practice, the nurse educator’s (a member of university staff) and the mentor’s (an experience clinical nurse) incompetence as well as a defensive, concealing and blaming work envıronment were among the essential challenges to involve student into practıce safety [49, 68]. The insufficient support from nurse educator was reflected in the encouragement to do things beyond their scope of practice or inconsistent with the student’s ethics and theory [69]. Further barriers appear after nursing students have realised that their mentors do not allocate enough time to teach patient safety or to assess them faithfully and adequately[49]. The dominant attitude of “getting the work done” and “following the rules” in the ward discourage students from questioning the procedures as they wish to conform rather than challenge the practices [40, 70]. Students just do routine work without compliance with the standards of caring principles and patient-centered approach [68]. This approach is not appropriate in the structure of system-wide patient safety knowledge.

The above consideration has been supported by the findings of this study and earlier studies. Students valued learning about patient safety on the technical aspects higher than the sociocultural aspects in both settings. The findings are consensus to other studies [44–46]. That indicates that a system–based approach is not realised by the students during their education [42, 51], as they are not engaged in learning the organisational strategies and systems in the clinical setting [39, 42]. This is an alarming finding as national and international reports address patient safety on a system-wide basis [13, 30, 71]. Nursing students enter the profession with an idealised view of nursing, which declines with increasing clinical exposure, as reality does not meet their expectations and their ideas related to professionalism (caring and compassion) [70, 72, 73], respectıvely and the confıdence in learning about sociocultural aspects [37, 44].

 The least agreement in this study in both settings was found in teamwork among all dimensions across countries and among years of study, while effective communication was valued higher in both settings. This weakness in interprofessional teamworkıng knowledge is supported with previous studies in Canada [38, 67], Finland [50, 51], Korea [61], Saudi Arabia [74], USA [23, 75] and Australia [44]. The findings support Cresswell et al., (2013) conclusion that students have been taught about safety in isolation from healthcare students of different disciplines. However, the suggestion “Interprofessional team training of nurses, physicians, and other health care providers should begin when they are students and proceed throughout their careers”[76]. Team culture was considered a strong influence in students’ decision on whether to speak up or remain silent and is reflected in the relationship with their mentor and other team members [39, 68] and was valued as a strong ethical responsibility to prevent errors [48]. Effective interprofessional collaboration has been found to prevent adverse patient safety events [68] as it challenges any gaps or misconceptions about the role of each discipline and responsibility in problem solving [77–79].

This comparative study also revealed that Cypriot students were more positive about what they have learned about patient safety issues than Greek students for all dimensions and in both settings. Sımılarly, former comparative studies [50, 51] indicated dıfferences between British and Finnish students, wıth British students obtaining higher knowledge in both settings and appreciating knowledge, skills, and attitudes on patient safety issues more than Finnish students did. These perceived differences reflected less training of skills in patient safety incidents received by the Finnish students. This is either due to the fact that England-UK patient safety work was ahead of Finland (i.e.introducing guidelines for patient safety since 2004) or because Finnish students’ experience focused on the traditional approach of blaming the individual [58]. Adding to these explanations, in the current study the perceived differences might be attributed to historical and political factors; for example, nursing education in Cyprus has been influenced to a great extent by the British colonial [59]. Other possible reasons are the different style of clinical supervision model used (i.e. mentor), the status of nurse educator ( i.e. employed or not by the university) and the status of students in clinical practice (i.e. supernumerary)[80, 81].

On the other hand, Greece has been in a profound economic crisis since 2010, which has affected the health care system and education [82]. Today, Greece faces significant nursing staff shortage as the ration of nurses per 1000 ınhabitants is the lowest in the EU [83]. Nursing understaffıng ın assocıated with the fact that Greece faces the unusual situation of having more medical doctors than nurses, in a ration of one nurse per doctor [84, 85] and almost half of the faculty members are medical doctors. This reality has consequently led to a medically-domınated care system in which the majority of care decisions are made by doctors, lımıtating nurses’ scope of practice. Besides, the negative clinical, educational learning opportunities may be partly related to the presence of assistant nurses to perform nursing duties which are incongruity to the respective training they have attained [85]. As a consequence, the environment ıs anything but ideal for learnıng about safe practıce having in mind that students’ professional socialisation in clinical learning is deeply influenced by observing assistant nurses [39]. Under this sight, it is admitted that poor caring is caused by the work system that does not allow nurses to perform to the best of their abilities [70]. In a broader view, the perceived differences may be associated to cultural diversity [51], the lack of consensus or clarity as to specific patient safety learning outcomes at European directives, curriculum and guidelines,[14] and the universities’ autonomy to accept new branches of nursing education [83].

As far as the years of study are concerned, progress in nursing students’ knowledge about patient safety tended to remain stable or increased across the years in both settings in all dimensions. This finding is consistent with Alquwez et al. [74] study. According to Benner [86], knowledge is embedded in expertise which develops through experience and exposure to clinical situations. However, inconsistency in this finding was found with the score declining across the years [37, 44, 65, 66]. This can either suggest that first-year students have less insight into their skills and abilities or that the more experienced ones have a better understanding of what patient safety is about and what is needed to ensure they practice in a ‘safe’ manner [44].

As for students’ perceptions of how broader patient safety issues were addressed, the students agreed upon the fact that their scope of practice is not clear. Early learning of the various healthcare professionals’ tasks and the scope of the role of nursing students is essential for them in accepting responsibility for their actions, be able to reflect on and internalise their clinical experiences, and learn to collaborate as a team member [87]. Faculty should clearly state to students what is expected from the clinical experience [70] and ascertain that nurse educators ensure assessment and learning curriculum outcomes achievement [88].

## Limitations

Data were collected by using a self-reported questionnaire where students may have overestimated or underestimated their awareness of patient safety knowledge. Also, data in Greece were collected from only one university in contradiction with Cyprus, which all universities were included. So, the transferability of the results needs to be carefully considered. Also, comparisons included students of different academic years without studying the progress of a single group of students over the years. Therefore, a longitudinal study covering the first to fourth-year period is recommended. Finally, students’ perception of knowledge to the clinical environment provides only a general view as questions did not refer to a specific clinical setting.

## Conclusions

The critical examination of the significant difference between class and clinical education indicate that the students perceived a gap between class and clinical practice. The view that patient safety competency cannot be acquired easily in a classroom alone provides an account to nurse educators’ endeavours to promote a dynamic interaction of knowledge among classroom and clinical practice and alert students to the realities of healthcare systems.

This study showed teamwork as the less covered competency in nursing education. Α clear description of the scope of the role of nursing students and understanding the different healthcare professionals’ roles and responsibilities are essential for effective teamwork, collaboration and enhancement of safer care by promoting understanding of different roles and responsibilities. These results indicate the lack of specific patient safety competencies in nursing students. These differences in students’ perceived competencies of patient safety, among two European Greek-speaking countries, clearly show that there is still inconsistency among patient safety education approaches and assessment methods despite the attempts to standardise nurse regulation and registration practices across Europe.

The impact of national patient safety efforts and guidance to nursing curricula and the influence of years of study on the patient safety competence of nursing students on the specific clinical environment needs to be further studied. This information can be used to update and design an international framework about the patient safety nursing curriculum adapted to national and cultural specificities as well as the students’ learning objectives. The “…*development it would seem that more than a series of statements relating to patient safety is required*“(p.138)[14].

## Data Availability

Data, to some extent, are presented within the manuscript. Authors do not wish to share their data because these data analysis are part of a PhD thesis which is still in progress. However, additional data can be available from the corresponding author on reasonable request after the completion of the thesis. All dataset wıll be submitted to the lıbrary of the Cyprus Unıversıty of Technology along with my thesis.

## Abbreviations

H-PEPSS: Health Professional Education Patient Safety Survey
